# Post-transcriptional control of endogenous retroviruses by L1td1 suppresses totipotency acquisition in pluripotent stem cells

**DOI:** 10.1038/s41421-025-00864-3

**Published:** 2026-01-20

**Authors:** Yi Wu, Yang Liu, Yile Huang, Zhihong Hao, Wenxin Li, Yukun Li, Maolei Zhang, Linpeng Li, Dajiang Qin, Keshi Chen, Xingguo Liu

**Affiliations:** 1 Institute of Development and Regeneration, Guangdong Provincial Key Laboratory of Stem Cell and Regenerative Medicine, Guangdong-Hong Kong Joint Laboratory for Stem Cell and Regenerative Medicine, GIBH-CUHK Joint Research Laboratory on Stem Cell and Regenerative Medicine, GIBH-HKU Guangdong-Hong Kong Stem Cell and Regenerative Medicine Research Centre, China-New Zealand Joint Laboratory on Biomedicine and Health, Guangzhou Institutes of Biomedicine and Health, Chinese Academy of Sciences, Guangzhou, Guangdong China; 2 University of Chinese Academy of Sciences, Beijing, China; 3 Centre for Regenerative Medicine and Health, Hong Kong Institute of Science & Innovation, Chinese Academy of Sciences, Hong Kong, SAR China; 4 Frontiers Medical Center, Tianfu Jincheng Laboratory, Chengdu, Sichuan China; 5 State Key Lab of Respiratory Disease, Joint School of Life Sciences, Guangzhou Medical University; Guangzhou Institutes of Biomedicine and Health, Chinese Academy of Sciences, Guangzhou, Guangdong China; 6 Guangdong Engineering Research Center of Early Clinical Trials of Biotechnology Drugs, The Fifth Affiliated Hospital, Guangzhou Medical University, Guangzhou, Guangdong China

**Keywords:** Stem cells, Cell biology

Dear Editor,

Early mammalian embryogenesis begins with a fertilized egg and zygotic genome activation (ZGA), the switch from maternal to zygotic control. In mouse two-cell (2C) embryos, ZGA is characterized by the transient activation of murine endogenous retrovirus-L (MERVL) and 2C-specific genes, such as *Zscan4*^1^. As development proceeds, expression of these genes declines as cells transition from a totipotent state, capable of producing all lineages, including extra-embryonic tissues, to a pluripotent state restricted to the three germ layers. Totipotency and pluripotency are dynamic cellular states central to development and hold great promise for both fundamental research and clinical applications in regenerative medicine^1^. Embryonic stem cells (ESCs) derived from the inner cell mass of blastocysts exhibit pluripotency, and a rare subpopulation termed two-cell-like cells (2CLCs) spontaneously emerges, recapitulating key molecular features and developmental potentials of 2C-stage embryos^2^. The molecular mechanisms governing ZGA and the transitions between pluripotency and totipotency have been increasingly elucidated across multiple regulatory layers, particularly at the transcriptional, epigenetic, and metabolic levels^1,3,4^. Recent studies reported that spliceosomal repression reprograms both human and mouse pluripotent stem cells (PSCs) toward a totipotent state^5^, suggesting an unexpected layer of post-transcriptional regulation. However, the precise post-transcriptional mechanisms governing pluripotency-totipotency transitions remain unclear.

RNA-binding proteins (RBPs) mediate post-transcriptional control of mRNA stability, localization, and translation, thereby influencing stem cell fate. Long interspersed nuclear element 1 (LINE1)-type transposase domain containing 1 (*L1td1*) is the only domesticated protein-coding gene almost entirely derived from the LINE1 (L1) retroelement^6^. L1td1 is highly expressed in PSCs and has been reported to be essential for maintaining pluripotency in human cells^7^. Intriguingly, evolutionary analyses suggest that L1td1 originated under positive selection in primates and rodents and was later co-opted into pluripotency networks^6^. Recent studies further reveal that L1td1 interacts with ancestral L1 ORF1p to facilitate L1 retrotransposition in cancer cells^8^. Despite links to transposable elements (TEs) and pluripotency, L1td1’s role in totipotency remains unexplored.

To investigate the potential role of L1td1 in embryonic development, we analyzed its expression across different developmental stages from single-embryo RNA-seq and proteomic datasets^9,10^. *L1td1* peaks at the late 2C stage when *Zscan4c* declines, and rises again in the inner cell mass, a trend also reflected at the protein level (Fig. 1a; Supplementary Fig. S1a), suggesting that L1td1 may play roles in both exit from totipotency and maintenance of pluripotency. We also assessed L1td1 protein expression in mouse embryonic fibroblasts (MEFs), ESCs, and 2CLCs induced by Dux. The results showed that the expression of L1td1 was high in ESCs but low in MEFs and 2CLCs (Fig. 1b). We then knocked down *L1td1* in ESCs harboring the 2C reporter MERVL::tdTomato and observed an increase in the proportion of MERVL^+^ cells (Fig. 1c; Supplementary Fig. S1b), which was confirmed by flow cytometry analysis (Fig. 1d). Transcriptomic analysis revealed that *L1td1* knockdown upregulated the expression of totipotency genes, including *Zscan4* family and ZGA-associated endogenous retroelements, MERVL-int (internal) and MT2_Mm (MERVL long terminal repeat (LTR)) (Fig. 1e; Supplementary Fig. S1c). Gene set enrichment analysis (GSEA) indicated an upregulation of 2C-specific genes following *L1td1* knockdown (Fig. 1f). Finally, qPCR analysis confirmed the increased expression of totipotency genes (Fig. 1g). In vitro embryo culture showed that *L1td1* knockdown increased the proportion of embryos arrested at the 2C and 4C stages, indicating that L1td1 contributes to the transition from totipotency to pluripotency (Supplementary Fig. S1d).

**Fig. 1:**
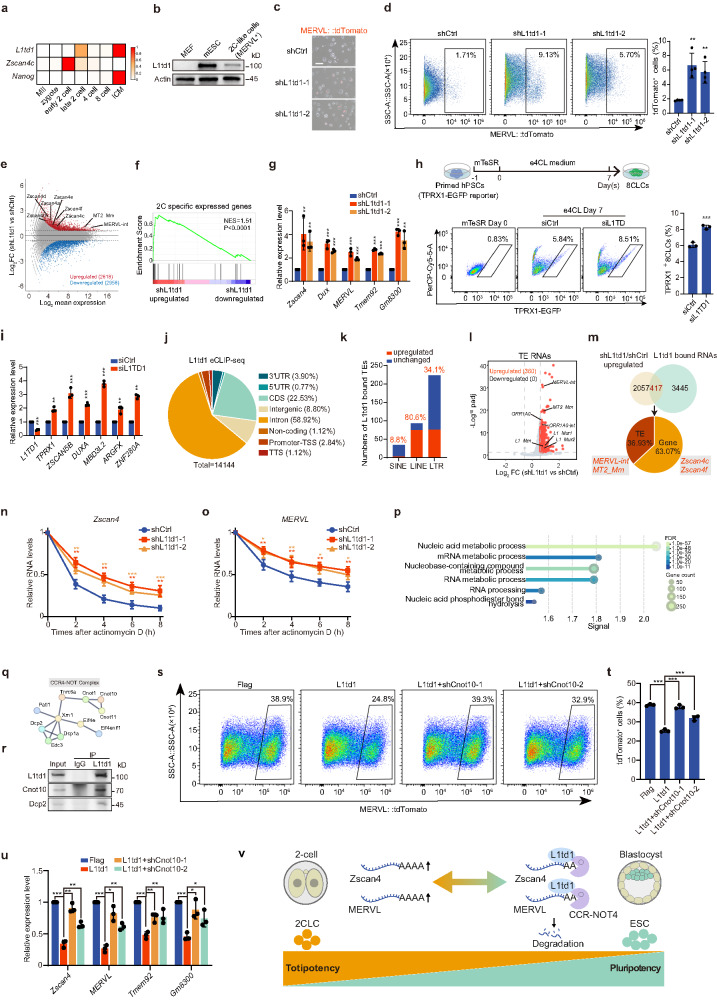
L1td1 suppresses totipotency acquisition in PSCs by regulating endogenous retroviruses. **a** mRNA expression of *L1td1* and stage-specific marker genes *Zscan4c* (2C) and *Nanog* (inner cell mass of blastocyst) at various developmental stages analyzed from published RNA-seq data. **b** Western blot analysis of L1td1 expression in MEFs, mESCs, and MERVL^+^ 2CLCs. **c** Phase contrast and fluorescence microscope images of mESCs with MERVL::tdTomato reporter transduced with shRNA control (shCtrl) or shRNA against *L1td1* (shL1td1-1 and shL1td1-2). Scale bar, 250 μm. **d** Left, flow cytometry analysis of 2CLCs (MERVL^+^) in mESCs transduced with shCtrl or shL1td1. Right, quantification of the percentage of 2CLCs in flow cytometry analysis. Data are mean ± SD, two-tailed unpaired *t*-tests, *n* = 3 biological replicates. **P < 0.01. **e** MA plot showing upregulation of 2C markers MERVL and *Zscan4s* in mESCs upon *L1td1* knockdown. **f** GSEA analysis showing upregulation of 2C-specific genes in mESCs upon *L1td1* knockdown. **g** qPCR analysis of the indicated 2C marker genes in mESCs transduced with shCtrl or shL1td1. Data are mean ± SD, two-tailed unpaired *t*-tests, *n* = 3 biological replicates. **P < 0.01, ***P < 0.001. **h** Top, schematic depicting the generation of 8CLCs from primed PSCs with direct e4CL medium. Bottom, flow cytometry and quantification analysis of 8CLCs (TPRX1^+^) in hPSCs transfected with siCtrl or siL1TD1. Data are mean ± SD, two-tailed unpaired *t*-tests, *n* = 3 biological replica*t*es. ***P < 0.001. **i** qPCR analysis of the indicated 8C marker genes. Data are mean ± SD, two-tailed unpaired *t*-tests, *n* = 3 biological replicates. **P < 0.01, ***P < 0.001. **j** Genomic distribution of L1td1 eCLIP-seq peaks (Fold enrichment **>**16 and *P* < 0.001). **k** Numbers of different types of TE RNAs bound by L1td1 and their expression changes upon *L1td1* knockdown. **l** Volcano plot showing the changes in TE RNA expression following *L1td1* knockdown. **m** Top, venn diagram showing the overlap between RNAs upregulated by shL1td1 and L1td1-bound RNAs. Bottom, pie charts illustrating the proportions of upregulated genes and TEs within the overlap. **n**, **o** RNA stability assay showing the relative RNA levels of *Zscan4* (**n**) and MERVL (**o**) at 0 h, 2 h, 4 h, 6 h, and 8 h after Actinomycin D treatment in mESCs transduced with shCtrl or shL1td1. Data are mean ± SD, two-tailed unpaired *t*-tests, *n* = 3 biological replicates. *P < 0.05, **P < 0.01, ***P < 0.001. **p** L1TD1-interacting proteins were identified by taking the intersection of two biological replicate IP-MS experiments, filtered through the CRAPome database. **q** GO analysis showing L1td1-interacting proteins. **r** Endogenous co-immunoprecipitation analysis of L1td1-interacting proteins. **s** Flow cytometry analysis of 2CLCs (MERVL^+^) in mESCs undergoing Dux-mediated 2C-like transition, with L1td1 overexpression, or L1td1 overexpression combined with *Cnot10* knockdown. **t** Quantification of the percentage of 2CLCs in (**s**). Data are mean ± SD, two-tailed unpaired *t*-tests, *n* = 3 biological replicates. ***P < 0.001. **u** qPCR analysis of the indicated 2C marker genes. Data are mean ± SD, *t*wo-tailed unpaired *t*-tests, *n* = 3 biological replicates. *P < 0.05, **P < 0.01, ***P < 0.001. **v** Model depicting the regulation of *Zscan4* and MERVL RNA s*t*ability by L1td1 through the CCR4–NOT pathway in regulating pluripotency and totipotency.

To investigate whether L1TD1 regulates totipotency in human cells, we knocked down *L1TD1* in primed human PSCs harboring the 8C reporter TPRX1-EGFP and induced their conversion into totipotent 8-cell-like cells (8CLCs) using the e4CL medium^11^. *L1TD1* knockdown increased the proportion of TPRX1^+^ cells and upregulated totipotency-associated genes (Fig. 1h, i), indicating a conserved function in both human and mouse cells.

To identify L1td1 targets, we performed enhanced crosslinking and immunoprecipitation sequencing (eCLIP-seq) in ESCs and found 14,144 binding peaks (Fig. 1j). Gene Ontology (GO) enrichment analysis of genes directly bound by L1td1 and upregulated upon its knockdown revealed a strong association with processes related to cell fate determination and early development (Supplementary Fig. S2a). Notably, we observed that a substantial proportion of L1td1-bound peaks are predominantly located in intergenic and intronic regions (Fig. 1j). L1td1 preferentially bound LINE and LTR TE RNAs rather than short interspersed nuclear element (SINE), and these RNAs were mostly upregulated (Fig. 1k). Moreover, most of the TE RNAs were upregulated upon *L1td1* knockdown, including ZGA-specific TEs such as MERVL-int, MT2_Mm, ORR1A0, and ORR1A0-int (Fig. 1l). Given that MT2_Mm and related elements function as alternative promoters initiating transcription, their upregulation likely reflects activation of the broader MERVL-LTR-driven totipotency-related transcriptome. Further analysis of RNAs bound by L1td1 and upregulated after its knockdown revealed that, in addition to totipotency genes like *Zscan4c* and *Zscan4f*, 36.9% of these RNAs were TE RNAs, including the totipotency-specific MERVL-int and MT2_Mm (Fig. 1m; Supplementary Fig. S2b, c). Actinomycin D assays showed that *L1td1* knockdown reduced degradation of *Zscan4* and TE-derived transcripts, including MERVL-int, MT2_Mm, and ORR1A0-int, indicating that *L1td1* knockdown enhances their stability (Fig. 1n, o; Supplementary Fig. S2d).

Notably, *L1td1* knockdown also increased L1 RNA levels (Fig. 1l; Supplementary Fig. S2e). Although L1 has been reported to repress the 2C program^12^, other studies show elevated L1 in conditions promoting 2C-like states, implying context- or dosage-dependent effects. Interestingly, we also identified Smarca5, an ISWI family chromatin remodeler previously reported to promote the 2C-like state^13^ among L1td1-bound transcripts upregulated upon *L1td1* knockdown. The RNA stability of *Smarca5* was increased upon *L1td1* knockdown (Supplementary Fig. S2f), suggesting that Smarca5 may act as an additional downstream mediator contributing to the 2C-like transition.

In human PSCs, *L1TD1* knockdown increased stability of *ZSCAN4* and HERV-K (two known markers of human totipotent-like state) transcripts but not HERV-H transcripts (Supplementary Fig. S3a–c), indicating that L1TD1 modulates a similar subset of RNAs in human and mouse cells and supporting a conserved post-transcriptional regulatory mechanism.

To investigate how L1td1 regulates RNA stability, we conducted immunoprecipitation followed by mass spectrometry (IP-MS) to identify its interacting proteins, which revealed that its interactors are primarily associated with RNA metabolism pathways (Fig. 1p; Supplementary Fig. S4a). Importantly, many of these interactors are components of the CCR4–NOT complex (Fig. 1q), suggesting that L1td1 may recruit the CCR4–NOT complex to facilitate RNA degradation. Endogenous co-immunoprecipitation confirmed the interaction of L1td1 with components of the CCR4–NOT complex, including Dcp2 and Cnot10 (Fig. 1r). Overexpression of L1td1 inhibited the Dux-induced 2CLC transition, which was largely reversed by *Cnot10* knockdown, as shown by the percentage of MERVL^+^ cells and expression of totipotency genes (Fig. 1s–u; Supplementary Fig. S4b). In addition, CCR4–NOT subunits display distinct temporal expression patterns during early embryogenesis, with *Cnot10* onset at the late 2C stage, consistent with sequential roles in maternal mRNA clearance and exit from totipotency (Supplementary Fig. S4c). Together, these results indicate that L1td1 recruits the CCR4–NOT complex to promote degradation of *Zscan4* and MERVL RNAs, thereby restricting acquisition of totipotency.

In summary, our study reveals a previously unrecognized function of the RBP L1td1 as a primary gatekeeper restricting the acquisition of totipotency in both human and mouse PSCs. L1td1-mediated RNA degradation safeguards pluripotency by eliminating totipotency-associated transcripts of *Zscan4* and endogenous retrovirus MERVL. Loss of *L1td1* stabilizes these RNAs, thereby promoting reversion to a totipotent state. Mechanistically, L1td1 recruits the CCR4–NOT complex to degrade these target RNAs, revealing a post-transcriptional mechanism for silencing the totipotency program. Our findings reveal a sophisticated post-transcriptional mechanism that silences TE RNAs in cell fate decisions and propose a strategy to acquire totipotent cells for xenogeneic organ generation in regenerative medicine.

TEs are crucial for early embryonic development, yet their post-transcriptional regulation remains unclear. Our research fills this gap by demonstrating that in PSCs, the RNA stability of TEs, including MERVL and L1, is controlled by the RBP L1td1 through the CCR4–NOT pathway. By maintaining appropriate levels of TE-derived transcripts, L1td1 safeguards pluripotency and suppresses the reversion to a totipotent state. These results identify a post-transcriptional mechanism that fine-tunes developmental potency and offer new insights into how RNA-binding proteins orchestrate early embryonic development, which may facilitate the generation of totipotent cells and their application in regenerative medicine.

While recent studies have highlighted the importance of RNA decay and modification in ERV suppression at the post-transcriptional level^14,15^, our study identifies L1td1 as a novel regulator that collaborates with the CCR4–NOT complex to directly degrade MERVL transcripts, limiting totipotency. This mechanism provides fresh evidence for post-transcriptional control of MERVL and reveals a new functional role for L1td1 in modulating cellular potency through TE RNA dynamics. Unlike previously described pathways, which predominantly involve epigenetic silencing or *N*^6^-methyladenosine (m^6^A)-mediated decay, L1td1-driven degradation offers a distinct route to prevent pluripotency reversion to totipotency. These insights deepen our understanding of how RBPs govern developmental transitions and highlight a previously unrecognized layer of MERVL regulation. Future investigations into the interplay between L1td1, m^6^A modifications, and other post-transcriptional factors will be essential to unravel the full complexity of TE regulation in embryogenesis. Moreover, we also identified several translational regulators and P-body-associated proteins as L1td1 interactors, suggesting that L1td1 may influence mRNA translation or sequestration, which warrants further investigation.

In addition, we provide evidence that L1td1, encoded by the only known protein-coding gene domesticated from a L1 retroelement, directly binds to and influences L1 RNA abundance. This supports a model in which domesticated transposon-derived proteins may repress evolutionarily younger retroelements at the post-transcriptional level. Notably, eCLIP-seq and motif analyses revealed that L1td1 preferentially binds discrete RNA motifs (GAGCGUC, GAAGAGC, CGUGUAG, and GAGAGAA), forming stem-loop or hairpin structures, enriched in totipotency and ZGA genes (Supplementary Fig. S5a–d). Phylogenetic analysis showed that L1td1 is highly conserved across mammals, indicating lineage-conserved RNA-binding functions (Supplementary Fig. S5e–g). By coupling transposon silencing with cell fate decisions, L1td1 may serve as a critical regulatory nexus, integrating the maintenance of genome stability with the dynamic control of developmental potential.

## Supplementary information

Supplementary information

## Data Availability

All data presented are available in the main text or the Supplementary information. The raw sequencing data reported in this paper have been deposited in the Genome Sequence Archive in the National Genomics Data Center, China National Center for Bioinformation (CRA025087 and CRA030513), which are publicly accessible at https://ngdc.cncb.ac.cn/gsa.
