# Supplementary material for: Post-transcriptional control of endogenous retroviruses by L1td1 suppresses totipotency acquisition in pluripotent stem cells
Source: Cell Discov. 2026 Jan 20;12:7. doi: 10.1038/s41421-025-00864-3 (PMC12820274; doi:10.1038/s41421-025-00864-3)
Supplement: Supplementary file 1 — Supplementary information [file 41421_2025_864_MOESM1_ESM.pdf]

Supplementary Information for

Post-transcriptional control of endogenous retroviruses by L1td1 suppresses totipotency acquisition in pluripotent stem cells

Authors: Yi Wu[1,2,7]*, Yang Liu[1,3,7], Yile Huang[2,7], Zhihong Hao[1], Wenxin Li[1,3], YuKun Li[1,3], Maolei Zhang[4], Linpeng Li[5], Dajiang Qin[6], Keshi Chen[1], Xingguo Liu[1,2,5]*

Affiliations:

[1]Institute of Development and Regeneration, Guangdong Provincial Key Laboratory of Stem Cell and Regenerative Medicine, Guangdong-Hong Kong Joint Laboratory for Stem Cell and Regenerative Medicine, GIBH-CUHK Joint Research Laboratory on Stem Cell and Regenerative Medicine, GIBH-HKU Guangdong-Hong Kong Stem Cell and Regenerative Medicine Research Centre, China-New Zealand Joint Laboratory on Biomedicine and Health, Guangzhou Institutes of Biomedicine and Health, Chinese Academy of Sciences, Guangzhou, China

[2]Centre for Regenerative Medicine and Health, Hong Kong Institute of Science & Innovation, Chinese Academy of Sciences, Hong Kong SAR, China

[3]University of Chinese Academy of Sciences, Beijing, China

[4]Frontiers Medical Center, Tianfu Jincheng Laboratory, Chengdu, Sichuan, China.

[5]State Key Lab of Respiratory Disease, Joint School of Life Sciences, Guangzhou Medical University; Guangzhou Institutes of Biomedicine and Health, Chinese Academy of Sciences, Guangzhou, China

[6]Guangdong Engineering Research Center of Early Clinical Trials of Biotechnology Drugs, The Fifth Affiliated Hospital, Guangzhou Medical University, Guangzhou, China.

[7]These authors contributed equally

*Correspondence: Xingguo Liu (liu_xingguo@gibh.ac.cn); Yi Wu (wu_yi@gibh.ac.cn)

This PDF file includes

Materials and Methods

Supplementary Tables

Supplementary Figures

References

Materials and Methods

Cell culture

OG2 mESCs and MEFs were obtained from E3.5 and E13.5 embryos, respectively, carrying the *OCT4*-GFP transgenic allele as previously described[1, 2]. Feeder cells derived from ICR mouse embryonic fibroblasts treated with mitomycin. OG2 mESCs were cultured on 0.1% gelatin-coated plates in DMEM/high glucose supplemented with Fetal Bovine Serum (Gibco, 15%), GlutaMAX (Gibco, 100×), NEAA (Gibco, 100×), 0.1 mM 2-mercaptoethanol (Gibco), 1000 U/mL leukemia inhibitory factor, 3 μM CHIR99021 (Selleck) and 1 μM PD0325901 (Selleck). MEF and HEK 293T cells were cultured in DMEM/high glucose (Gibco) supplemented with 10% FBS (Gibco), GlutaMAX (Gibco, 100×), and NEAA (Gibco, 100×). 2CLCs were induced from OG2 mESCs with a MERVL::tdTomato reporter in the presence of 1 mg/mL doxycycline (selleck). Primed human PSCs (GZF2-iPS) were cultured in mTeSR medium (Stemcell Technologies) on plates coated with Matrigel (Corning). All animal experiment protocols were approved and conducted in accordance with guidelines of the Institutional Animal Care and Use Committee (IACUC, No. 2021059) of the Guangzhou Institutes of Biomedicine and Health, Chinese Academy of Sciences (GIBH, CAS). All experiments involving human pluripotent stem cell (hPSC) lines were conducted in accordance with the Declaration of Helsinki and approved by the institutional ethics committee of GIBH, CAS.

Induction of human 8CLCs

Human 8CLC were induced with direct e4CL methods as previously described[3]. Briefly, primed PSCs were dissociated into single cells and plated on feeders in mTeSR medium

supplemented with 10 μM Y-27632. After 24 h, medium was switched to e4CL and refreshed every day until day 5 or 7.

Plasmids and cell transfection

The coding sequences of Dux and L1td1 were cloned into pRlenti-EF1α−2A-Puro or PiggyBac vectors. shRNA constructs were inserted into the pLKO.1 lentiviral vector, with the corresponding shRNA sequences listed in Supplementary Table 1. Cells were transfected using Lipofectamine Stem Transfection Reagent (Thermo) or polyethylenimine (Polysciences). Lentivirus were produced in HEK293T cells and used to infect mESCs in the presence of polybrene (Beyotime), followed by puromycin selection (Selleck) for 2 days.

Flow cytometry

Cells were washed with PBS, dissociated into single cells using 0.25% Trypsin-EDTA, and collected by centrifugation. The cell pellet was resuspended in PBS containing 0.1% BSA, filtered through a cell strainer (BD Biosciences), and analyzed using an LSR Fortessa SORP flow cytometer (BD Biosciences). Data analysis was conducted with FlowJo v10.0.7.

Western blot

Whole-cell extracts were lysed with radioimmunoprecipitation (RIPA) buffer (Beyotime) supplemented with a protease inhibitor cocktail (Bimake). Proteins were analyzed by SDS-PAGE and then transferred to a PVDF membrane (Millipore). The membrane was incubated with primary antibodies and developed on X-ray film. The following antibodies were used: anti-Actin (HUABIO, ET1702-52, 1:5000), anti-L1td1 (Proteintech, 21528, 1:5000) and goat anti-rabbit (Kangchen, KC-RB-035, 1:5000)

Quantitative PCR (qPCR）

Total RNA was extracted using the EZ-press RNA Purification Kit (EZBioscience), and 4 μg of RNA was reverse transcribed into complementary DNA using HiScript II

reverse transcriptase (Vazyme). Gene expression levels were quantified using ChamQ SYBR qPCR Master Mix (Vazyme) and analyzed with the CFX-96 Real-Time PCR System (Bio-Rad). The primer sequences are provided in Supplementary Table 2.

Immunoprecipitation

Cells were lysed in NP-40 lysis buffer (Beyotime) containing protease inhibitor cocktail for 30 minutes at 4 °C. Normal rabbit IgG (Cell Signaling, 2729, 1:200) or anti-L1td1 antibody (Proteintech, 21528, 1:200) were conjugated to Dynabeads protein A and G (Thermo) for 4 hours at 4 °C in lysis buffer. The lysates were then incubated with the antibody-conjugated Dynabeads overnight at 4 °C. The beads were washed with lysis buffer for at least 5 minutes, repeated three times. The eluates were subsequently analyzed by western blot or mass spectrometry.

RNA stability assay

Cells were treated with 10 μg/ml Actinomycin D (Selleck) for 0, 2, 4, 6, or 8 hours, and RNA was extracted at the specified time points for subsequent qPCR analysis. RNA stability was assessed by measuring the relative amount of target mRNA remaining after transcription was inhibited by Actinomycin D.

RNA-seq

Total RNA was extracted using TRIzol. Libraries were constructed with the Illumina TruSeq RNA Sample Prep Kit according to the manufacturer's instructions. The experiments were conducted by Annoroad Gene Technology (Beijing, China). Clean data were analyzed using STAR software, and differentially expressed genes were identified with DESeq2. Gene Ontology analysis was performed using the DAVID database (https://david.ncifcrf.gov), with P values representing the modified Fisher's exact corrected EASE score.

eCLIP-seq and data analysis

A total of 1×10^7 mESCs were cross-linked with 254 nm UV light at 400 mJ/cm^2, lysed, and digested with RNase I (Thermo Fisher). The lysates were incubated with 10 µg of anti-L1td1 (Proteintech, 21528) conjugated to Dynabeads with protein A and G for 4 hours, followed by washing, end repair, and 3′ adaptor ligation. The protein-RNA complexes were eluted from the beads, resolved by denaturing gel electrophoresis, and transferred to a nitrocellulose membrane. Library preparation was performed by DIATRE Biotechnology (Shanghai, China) following the original protocol[21]. The data were trimmed for adapters using Cutadapt and then mapped against the full mouse genome (mm10) using STAR. PCR duplicates were removed with Picard (http://broadinstitute.github.io/picard/), and only read 2 was retained by SAMtools for subsequent cluster identification using CLIPper[4] (https://github.com/YeoLab/clipper). Peak-level input normalization was performed by overlapping the eCLIP reads of the sample with the paired SMInput sample to identify peak counts, from which fold-enrichment was calculated. Enrichment p-values were further determined using Chi-Square or Fisher's Exact Test.

Statistical analyses

The statistical tests used in this study are indicated in the respective figure legends. In general, data were analyzed by student unpaired t test to determine statistically significant effects (*$p < 0.05$, **$p < 0.01$, ***$p < 0.001$).

Supplementary Tables

Supplementary Table S1. shRNA and siRNA sequences used in this study

shRNA/siRNA sequences

shCnot10-1	TGCTGCATTGCTGCCAATAAA
shCnot10-2	TTGCCTGTCTCCAAGATATAA
shL1td1-1	GCGAAGTACCTGCATCCATGA
shL1td1-2	GCATTTGGAAGAGCGGATAGG
siL1TD1	GGUAAAUUAGACAACACUATT

Supplementary Table S2. qPCR primers used in this study

qPCR primers

Zscan4	GTCCTGACAGAGGCCTGCC	GAGATGTCTGAAGAGGCAAT
Dux	ACTTCTAGCCCCAGCGACTC	CCATGCTGCCAGGATTTCTA
MERVL-LTR	CTTCCATTCACAGCTGCGACTG	CTAGAACCACTCCTGGTACCAAC
Tmem92	GGGGCACACTCACCTTGAC	CAGCATTCCTTgACACAGCAT
Gm8300	TACTCACCAGGTCAATgCAGG	GTCCTGGCTCCTGATAGTTAC
Cnot10	CACGTCTGGAAATTATGATGCCT	AGCCGAGTGGACCTGATTCT
Actin	GGCTGTATTCCCCTCCATCG	CCAGTTGGTAACAATGCCATGT
18S	CTTAGAGGGACAAGTGGCG	ACGCTGAGCCAGTCAGTGTA
TPRX1	CAGACACCCAGTTATTCCCTCA	GCCCATAGAGTCATCCCCTTCT
ZSCAN5B	ATGGCTGCAAATTGGACACTC	AGGGTTCCTGTCGTGATTTCC
DUXA	GGGCAAGATCAACCTGGTGT	TGTGTAACTGAGAGGCGCTG
MBD3L2	CTTCCGGAGGCCGGTGACAA	TGCGGCTTCTCCAGGTGCTC
ARGFX	AGCAATACGGAGAAGGCATAAAG	GGTCGAGTCTCAAAGCTAGTTTC
ZNF280A	CCCCAGGCTCAAAGTCAAGAA	CGGGCATGATGGCTTTTGC
ACTB	CATGTACGTTGCTATCCAGGC	CTCCTTAATGTCACGCACGAT
HERV-K	GCCATCCACCAAGAAAGCA	AACTGCGTCAGCTCTTTAGTTGT
HERV-H	TTCACTCCATCCTTGGCTAT	CGTCGAGTATCTACGAGCAAT
Smarca5	TGCAAACTGACCGGGCAAATA	TCGCCAACGGATAGTAAGTTCT

Supplementary Figures

Supplementary Figure 1

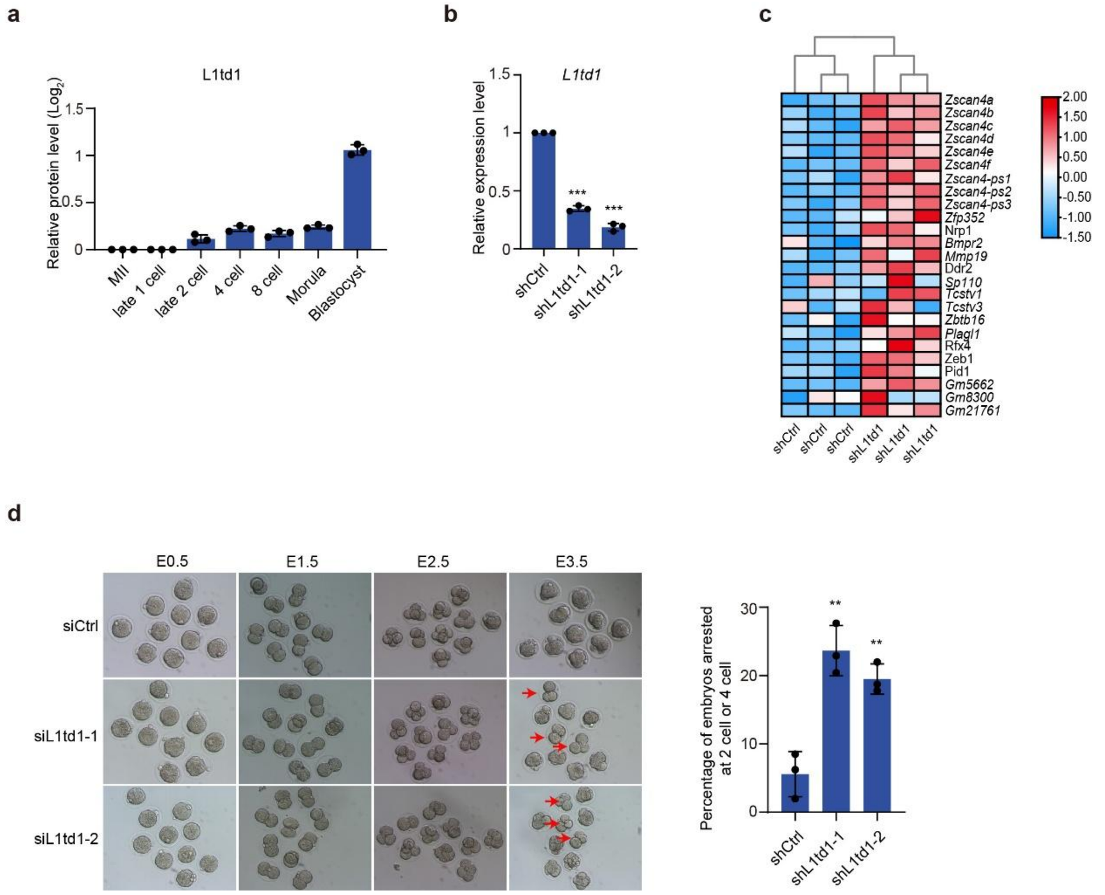

Supplementary Figure S1. L1td1 suppresses totipotency acquisition in mouse pluripotent stem cells

a. Protein level of L1td1 obtained from single-cell embryonic proteomic data.

b. qPCR analysis of L1td1 expression in mESC transfected with shRNA control (shCtrl) or shRNA against *L1td1* (shL1td1-1 and shL1td1-2). Data are mean ± s.d., Two-tailed unpaired t-tests, n = 3 biological replicates. ***P < 0.001.

c. Heatmap showing the expression of 2C specific genes in mESCs transduced with shCtrl or shL1td1.

d. Developmental efficiency of embryos following zygotic injection of siCtrl or siL1td1. The proportions of embryos arrested at the 2-cell and 4-cell stages were quantified. Data are mean ± s.d., Two-tailed unpaired t-tests, n = 3 biological replicates. **P < 0.01.

Supplementary Figure 2

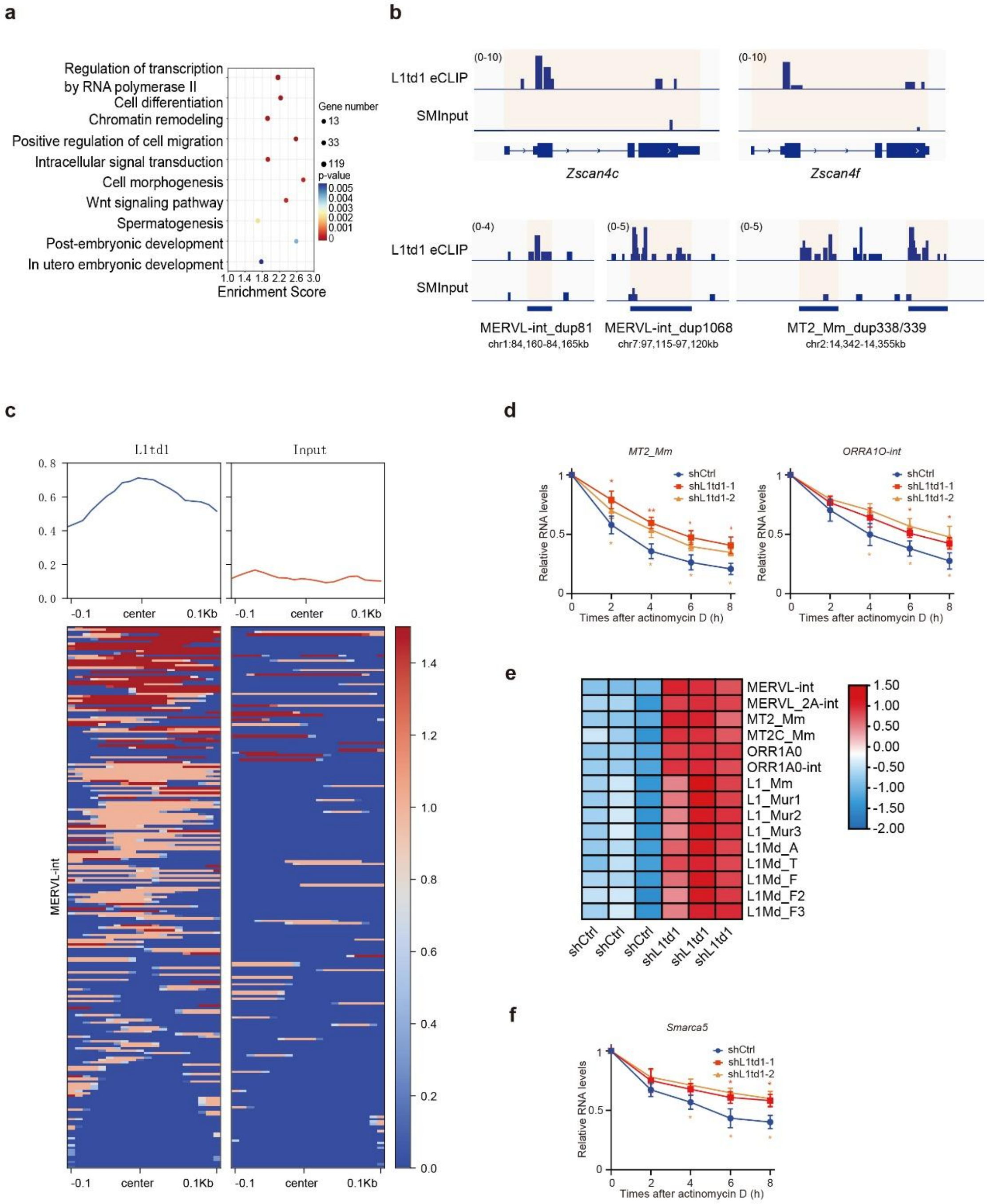

Supplementary Figure S2. L1td1 binds transcripts of totipotency-associated genes and endogenous retroviruses and promotes their degradation.

a. Gene ontology (GO) term analysis of genes bound and regulated by L1td1.

b. IGV showing L1td1 binding peaks at *Zscan4c/f*, *MERVL-int* and *MT2_Mm* loci.

c. Genomic views of L1td1 binding on *MERVL-int*.

d. RNA stability assay showing the relative RNA levels of *MT2_Mm* and *ORRA1O-int*

at 0, 2, 4, 6, and 8 hours after Actinomycin D treatment in mESCs transduced with shCtrl or shL1td1. Data are mean ± s.d., Two-tailed unpaired t-tests, n = 3 biological replicates. *P < 0.05,**P < 0.01.

e. Heat map showing representative endogenous retrovirus expression after L1td1 knockdown.

f. RNA stability assay showing the relative RNA levels of *Smarca5* at 0, 2, 4, 6, and 8 hours after Actinomycin D treatment in mESCs transduced with shCtrl or shL1td1. Data are mean ± s.d., Two-tailed unpaired t-tests, n = 3 biological replicates. *P < 0.05.

Supplementary Figure 3

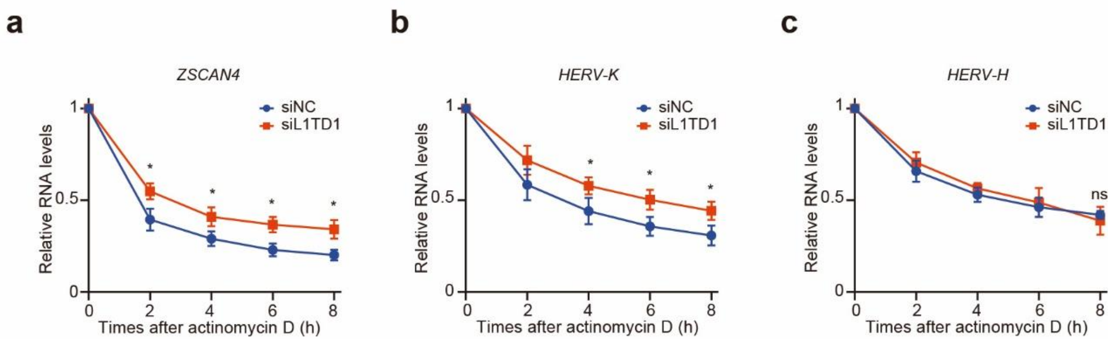

Supplementary Figure S3. L1td1 promotes degradation of *ZSCAN4* and *HERV-K* in human pluripotent stem cells.

a. RNA stability assay showing the relative RNA levels of *ZSCAN4*, *HERV-K* and *HERV-H* at 0, 2, 4, 6, and 8 hours after Actinomycin D treatment in human primed PSC transfected with siNC or siL1td1. Data are mean ± s.d., Two-tailed unpaired t-tests, n = 3 biological replicates. *P < 0.05.

Supplementary Figure 4

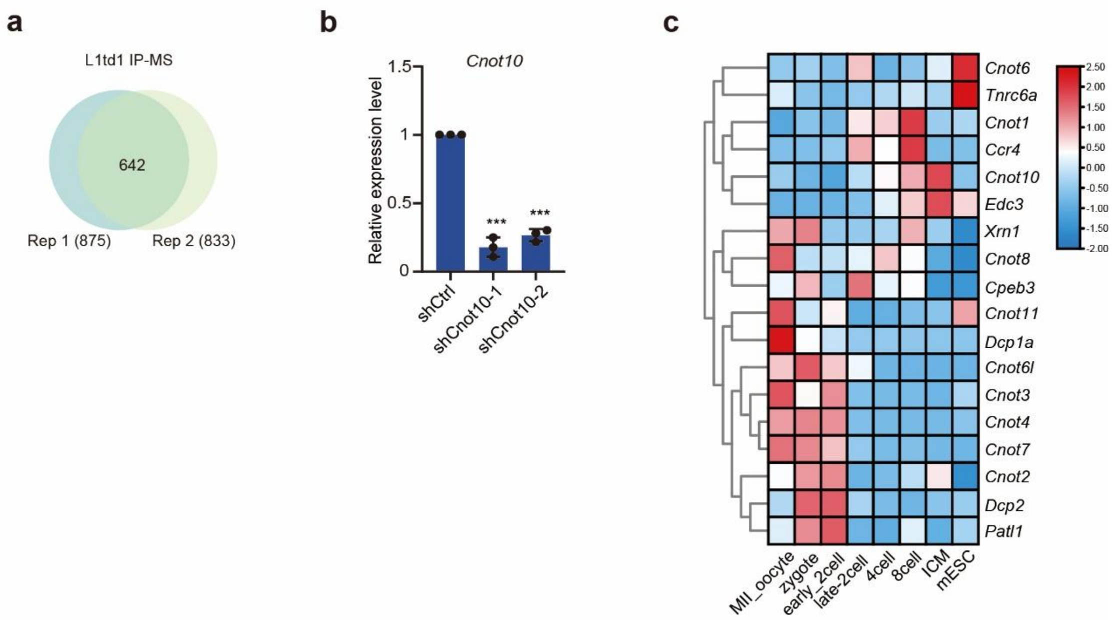

Supplementary Figure S4. L1TD1-interacting proteins identified by IP-MS.

a. L1TD1-interacting proteins were identified by taking the intersection of two biological replicate IP-MS experiments, filtered through the CRAPome database.

b. qPCR analysis of Cnot10 expression in mESCs transduced with shCtrl or shRNA against Cnot10 (shCnot10-1 and shCnot10-2). Data are mean ± s.d., Two-tailed unpaired t-tests, n = 3 biological replicates. ***P < 0.001.

c. Heatmap showing expression of CCR4-Not complex during early embryonic development.

Supplementary Figure 5

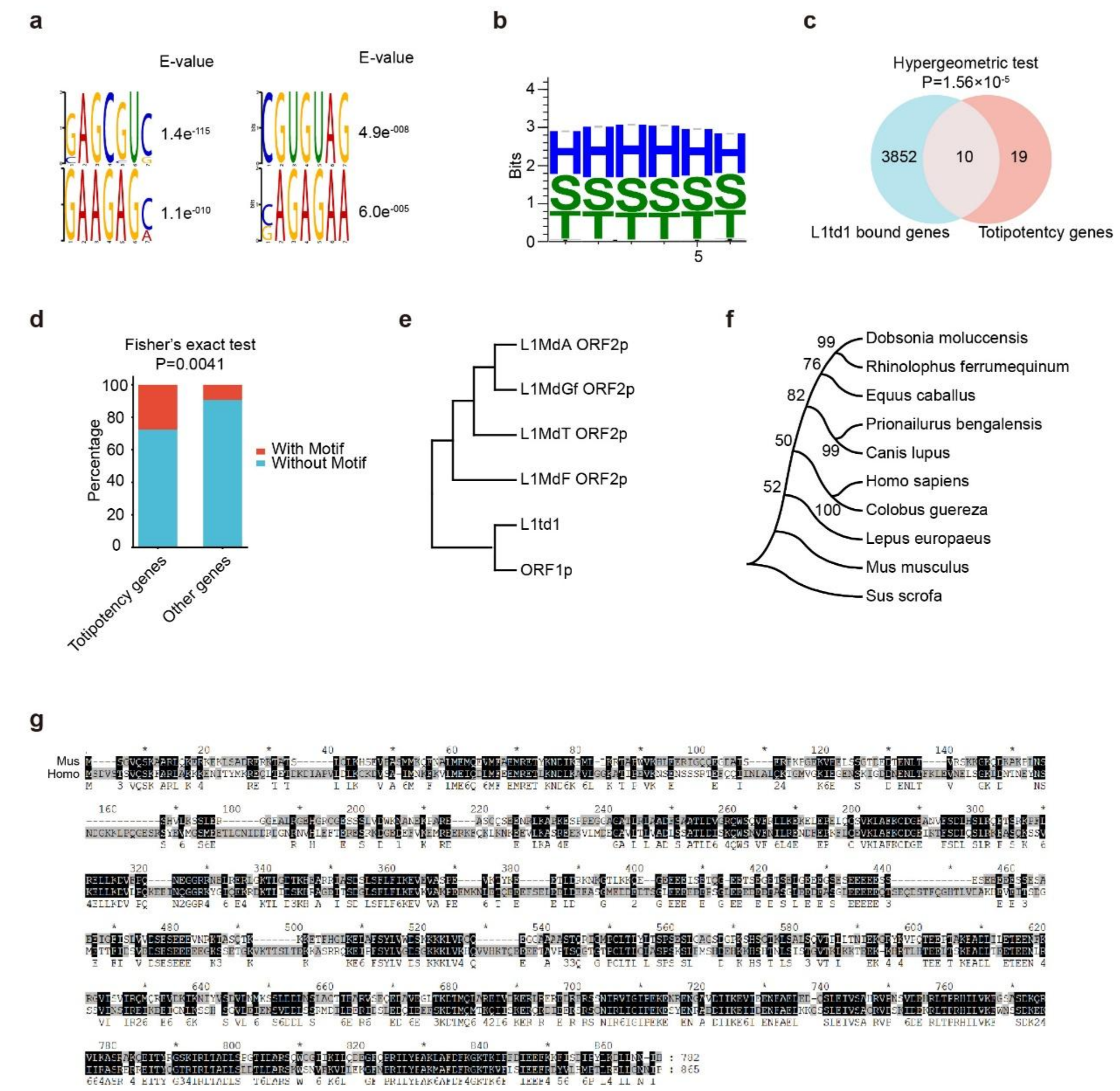

Supplementary Figure S5. Binding preference and evolutionary conservation of L1td1.

a. Sequence motifs enriched in eCLIP peaks for L1td1 binding.

b. Information content plot of the predicted RNA secondary structure within L1td1-binding regions.

c. Overlap between L1td1-bound genes and totipotency-related genes. A hypergeometric test indicates significant enrichment (P=1.56 × 10^{-5}).

d. Percentage of totipotency-associated genes and other genes containing the enriched motif. Fisher's exact test shows a significant association (P=0.0041).

e. Phylogenetic tree of protein sequences for L1td1 and transposases from other LINE-1 subfamilies, illustrating their evolutionary relationships.

f. Phylogenetic analysis of L1td1 orthologs across mammalian species, with bootstrap values indicated at branch points.

g. Multiple sequence alignment of L1TD1 proteins from mouse (Mus musculus) and human (Homo sapiens), with conserved regions highlighted.

References

1. Wu, Y. *et al.* Plin2-mediated lipid droplet mobilization accelerates exit from pluripotency by lipidomic remodeling and histone acetylation. *Cell Death Differ* **29**, 2316-2331 (2022).
2. Wu, Y. *et al.* Phospholipid remodeling is critical for stem cell pluripotency by facilitating mesenchymal-to-epithelial transition. *Sci Adv* **5**, eaax7525 (2019).
3. Mazid, M.A. *et al.* Rolling back human pluripotent stem cells to an eight-cell embryo-like stage. *Nature* **605**, 315-324 (2022).
4. Van Nostrand, E.L. *et al.* Robust transcriptome-wide discovery of RNA-binding protein binding sites with enhanced CLIP (eCLIP). *Nat Methods* **13**, 508-514 (2016).
